# Farewell

**DOI:** 10.4103/0970-0358.44918

**Published:** 2008

**Authors:** Mukund R. Thatte

**Affiliations:** Consultant Hand and Plastic Surgeon, Prof. of Plastic Surgery, Bombay Hospital Institute of Medical sciences, Editor, IJPS, Mumbai, India. E-mail: mthatte@vsnl.com

As I write my last editorial I continue to be amazed by the volume of work our colleagues produce and present. It could be the result of hard labour where a question which bothers the surgeon is answered by designing a meticulous study with appropriate ethical oversight, informed consent and proper controls. Some papers, however, are a result of a great idea one gets while mulling over a subject. They are like bright lights that change paradigms in our thinking.

We lead this issue with just such a paper by Prof. Mukund Jagannathan, who has put together bits and pieces from various accepted techniques on the TM joint and also cartilage transfer but has come out with an innovation which was pretty obvious but yet never really done before. We have incorporated his video on www.ijps.org to make our journal a truly multimedia, open access platform.

The paper by Prof. Vasco Senna Fernandes too is path breaking in that he has combined the ancient Chinese knowledge of medicine and modern aesthetic surgical thinking to create a new way of looking at marking and planning for aesthetic surgical procedures.

We also present you a remarkable case report by Dr Yogesh Bhatt and colleagues on one of the rarest anomalies in hand surgery. It is not only the authors who think so. An external reviewer and expert in Anatomy and embryology Prof. Bersu, has presented his comments in the invited discussion. The same unit also presents a rather inspiring model for public private partnership in the form of Project Muskan. We ought to replicate this round the country.

The world is increasingly turning to English as the lingua franca for scientific discourse. Existing journals simply did not have the space to do justice to the work now coming out from diverse regions beyond the traditional ‘west’. There is space for another English language journal to provide this platform. IJPS in its current avatar started out by filling this space. It helps that this journal is also the official journal of the Association of Plastic Surgeons of India, a body representing a country of a billion plus people. It was inconceivable that a country as large and as dynamic as today's India did not have a modern journal of Plastic Surgery.

We have today managed to make the IJPS completely in tune with the best of Modern Technology, both for manuscript submission and online publication. The participation from countries other than India has now grown to 30%, thus substantiating my earlier point. Our web readership is about 15,000 per issue. We are now indexed in multiple indices and finally from March 2009 we will get searched from Pubmed central as well. It must be noted that we have done this without compromising open access. I have always supported this movement and I am aware that my successor supports it as well. In Dr. Surajit Bhattacharya we now have a dynamic and scholarly new editor. He will bring in new perspectives, new ideas and above all fresh energy to carry on this task. I am confident that he will take the IJPS to new heights. I have immensely enjoyed being your editor for the last 6 years. It is time to step down.

During my tenure I have been supported tirelessly by the Editorial board, the Trustees and the Executive Council of APSI. Dozens of reviewers from around the world deserve my gratitude for their peer reviews which allowed us to maintain standards. Since last two years 3 deputy editors viz. Dr Milind Wagh, Dr. R. Srikanth and Dr. Wolfgang Huber have helped by taking over various tasks. I wish to thank all of them from the bottom of my heart for their help without which I would have never succeeded in completing my tasks. I wish to thank all the organisations whose financial support has helped keep us open access. Medknow publications led by Dr. D.K. Sahu have done yeoman service to India's medical publishing efforts and I wish to acknowledge their role in the success of IJPS. In the end I wish to acknowledge my wife Dr. Urmila Thatte for meticulously proof reading all the 14 issues that were published in my tenure.

Finally none of this was possible without the enthusiastic support and contributions of the readers and investigators from all over the world and I sincerely thank all of you. Farewell dear readers and best wishes to the new team at IJPS.

